# Progression of Retinal Ganglion Cell and Nerve Fiber Layer Loss in Spinocerebellar Ataxia 3 Patients

**DOI:** 10.1007/s12311-023-01634-1

**Published:** 2023-11-30

**Authors:** Anna Camós-Carreras, Marc Figueras-Roca, Marina Dotti-Boada, Rafel Alcubierre, Ricardo Pedro Casaroli-Marano, Esteban Muñoz, Bernardo Sánchez-Dalmau

**Affiliations:** 1 Ophthalmology Department, Seu Maternitat, Hospital Clínic de Barcelona, Universitat de Barcelona, Sabino de Arana 1, 08028 Barcelona, Spain; 2 Faculty of Medicine and Health Sciences, Universitat de Barcelona, Casanova 143, 08036 Barcelona, Spain; 3 Fundació Per La Recerca Biomèdica-IDIBAPS, Villarroel 170, 08036 Barcelona, Spain; 4 Neurology Department, Seu Villarroel, Hospital Clínic de Barcelona, Villarroel 170, 08036 Barcelona, Spain

**Keywords:** Machado-joseph disease, Spinocerebellar ataxia type 3, Optical coherence tomography, Retinal ganglion cell

## Abstract

Spectral domain optical coherence tomography (SD-OCT) allows noninvasive measurements of retinal neuron layers. Here, we evaluate the relationship between clinical features and anatomical SD-OCT measurements in patients with spinocerebellar ataxia type 3 (SCA3) and how they change with time. A retrospective review was conducted on SCA3 patients. Clinical variables such as disease duration, number of CAG repeats, and the Scale for the Assessment and Rating of Ataxia (SARA) score were correlated with SD-OCT measurements, including retinal nerve fiber layer (RNFL) thickness, ganglion cell complex (GCC) thickness, macular volume (MV), and central macular thickness (CMT). Seventeen SCA3 patients with an average follow-up of 44.9 months were recruited. Clinical features with significant baseline correlations with SD-OCT measurements included disease duration (CMT *r* =  − 0.590; GCC *r* =  − 0.585), SARA score (CMT *r* =  − 0.560; RNFL *r* =  − 0.390), and number of CAG repeats (MV *r* =  − 0.552; RNFL *r* =  − 0.503; GCC *r* =  − 0.493). The annual rate of change of the SARA score during follow-up was associated with that of both the MV (*r* =  − 0.494; *p* = 0.005) and GCC thickness (*r* =  − 0.454; *p* = 0.012). High disability (stages 2 and 3) was independently inversely associated with the annual change in MV (ß coefficient − 17.09; *p* = 0.025). This study provides evidence of an association between clinical features and objective anatomical measurements obtained by SD-OCT in SCA3 patients. MV and GCC thickness could serve as potential biomarkers of disease severity, as their rates of decrease seem to be related to a worsening in the SARA score. These findings highlight the potential of SD-OCT as a noninvasive tool for assessing disease severity and progression in SCA3 patients.

## Introduction

Spinocerebellar ataxia (SCA) is a heterogeneous entity that comprises a group of autosomal dominantly inherited neurological disorders mainly characterized by progressive ataxia. SCA results from the degeneration of cerebellar cells accompanied by degenerative changes in the brainstem and other parts of the central nervous system [1]. Among the various subtypes of SCA, SCA3, also known as Machado-Joseph disease, is the most prevalent form worldwide, affecting an estimated 1 to 4 individuals per 100,000. SCA3 presents with a multifaceted clinical profile characterized by progressive ataxia accompanied by nystagmus, ophthalmoparesis, and impaired speech and swallowing. Additionally, patients may exhibit symptoms such as parkinsonism, dystonic postures, chorea, peripheral neuropathy, and upper and lower neuron motor disturbances. Neuropathological findings included widespread degeneration, affecting cerebellar afferent and efferent pathways, pontine and *dentate nuclei*, as well as the cell bodies of the *substantia nigra*, subthalamic nucleus, *globus pallidus*, cranial motor nerve nuclei, peripheral nerves, pyramidal tracts, and anterior horn [2].

Despite its clinical prominence, SCA3, like many neurodegenerative disorders, lacks reliable biomarkers for tracking disease progression and assessing the efficacy of potential therapeutic interventions. While extensive clinical research has provided insights into the diverse clinical aspects of SCA3, there is an unmet need for a comprehensive and objective investigation into the direct structural neural changes that occur over time in this disease.

In many neurological diseases, central nervous system involvement can be assessed through the examination of eye fundus structures, such as the retinal nerve fiber layer (RNFL) of the optic nerve [3]. Optical coherence tomography (OCT) offers an objective and noninvasive imaging technique capable of quantitatively measuring eye tissue layers, including the RNFL and ganglion cell complex layer (GCC). Previous reports have explored RNFL and GCC changes in SCA patients, revealing mild reductions in layer thickness. These findings suggest that OCT measurements could serve as valuable biomarkers for assessing both clinical severity and disease progression [4, 5]. Moreover, photoreceptor degeneration can also be found in some forms of SCA (SCA7), preceding, accompanying, or following the onset of ataxic symptoms [6]. This has also been described in some recessive ataxias, such as Friedreich ataxia, which has been found to show afferent visual pathway damage. OCT may detect asymptomatic optic neuropathy, demonstrating a variable reduction in the RNFL in Friedreich ataxia patients [7]. Nonetheless, some neurodegenerative diseases, such as Parkinson’s and Alzheimer’s disease, also present with thinning of the RNFL and GCC [8–10].

Our study undertakes a retrospective cohort analysis of patients with SCA3, with the primary objective of examining quantitative changes in the RNFL and GCC using OCT. This investigation holds great significance, as it offers a pathway to discerning and quantifying structural neural changes that could serve as invaluable biomarkers for monitoring SCA3 progression and assessing the potential efficacy of future therapeutic strategies.

## Materials and Methods

Patients with clinically and genetically confirmed SCA3 were recruited from the Hospital Clínic de Barcelona between January 2020 and January 2021. Genetic diagnosis had previously been carried out in a clinical care setting (Genetic Service of Hospital Clinic de Barcelona) following standard procedures. SCA3 diagnosis was definitively established when genetic testing confirmed the presence of a CAG trinucleotide repeat expansion with more than 60 repeats in the ATXN3 gene on chromosome 14q24.3-q31 [11].

This study followed the ethical precepts of the Declaration of Helsinki (Fortaleza, Brazil, Oct 2013) and was approved by the local ethics committee (CEIm, HCB/2023/0674, Hospital Clinic de Barcelona). The use of personal data complied with local regulations (Law 05/2018). Retrospective data collection was undertaken for all patients, including neurological history, disease stage, age at onset, duration of the disease, and total follow-up. Exclusion criteria included a cause of visual loss other than SCA, any other optic neuropathy (glaucoma, optic nerve toxicity, etc.), and any macular disease and refractive defects (hyperopia higher than 5 optic dioptres and myopia higher than 5 optic dioptres) due to variability of RNFL thickness measurements in this population.

To assess disease severity, the Scale for the Assessment and Rating of Ataxia (SARA) was used [12]. The SARA score, the sum of the scores of the evaluation of eight quantitative features for gait, stance, sitting, speech disturbance, and limb kinetic functions, ranges from 0 (no ataxia) to 40 (most severe form of ataxia). The disease stage (stage 1, abnormal but independent gait; stage 2, support [cane, crutch] is needed to walk; stage 3, wheelchair dependency) of each patient was collected and binarized into low (stages 1 and 2) and high (stage 3) disability [13]. Each patient’s clinical history was retrospectively reviewed until the newest ophthalmologic evaluation (recruitment visit). A complete ophthalmologic examination was always performed, including best-corrected visual acuity (BCVA) with a Snellen chart, color vision (Ishihara’s test), ocular motility, slit-lamp biomicroscopy, intraocular pressure measurement, and indirect ophthalmoscopy. Spectral-domain OCT (SD-OCT; Cirrus HD, Carl-Zeiss Meditec) was performed under pupil dilation in all patients to measure the parapapillary RNFL thickness, central macular thickness (CMT), macular volume (MV), and macular GCC thickness. SD-OCT acquisition protocols included an Optic Disc Cube 200 × 200 and a Macular Cube 521 × 128, which were used to quantify the RNFL and GCC thicknesses via the commercial built-in segmentation software. SD-OCT acquisition protocols were carried out according to the criteria OSCAR-IB and APOSTEL 2.0 [14, 15]. Image analysis was performed using the manufacturer’s built-in software, and the results were compared with the device’s normality database. Automated segmentation accuracy was subsequently reviewed and manually corrected, if deemed necessary.

Core clinical variables included the BCVA (logMAR), central macular thickness (CMT, µm), macular volume (MV, mm^3^), overall RNFL thickness (µm), average GCC (µm), and minimum GCC (µm). The main outcome result was the change in these variables during follow-up, as well as the association of these clinical data with SCA3 disease characteristics (disease duration, number of CAG repetitions, and SARA score).

### Statistical Analysis

Absolute frequencies and percentages are used to describe categorical variables. Quantitative variables are described with the mean, standard deviation (SD), and range; changes in quantitative variables are reported as the mean percentage of change (%) and mean annual absolute rate of change. The Shapiro‒Wilk test was used to assess the normality of the distributions of the data. The paired samples *t* test, chi-squared test, or Fisher’s exact test was used to assess subject changes during follow-up. The correlation between the annual rates of change of SD-OCT features and the annual rate of change of the SARA score was analyzed by calculating the Pearson or Spearman correlation coefficient. A backward stepwise logistic regression model for the final follow-up disability stage was accordingly developed with variables with a significance level < 0.1 in the univariate analysis included as independent factors. A bilateral type I error of 5% was established. The statistical package STATA v.15.1 (StataCorp, College Station, TX, USA) was used for the analysis.

## Results

Seventeen SCA3 patients (34 eyes), including 10 females (58.8%), were included. A description of the cohort is summarized in Table 1. The mean (± SD) age at disease onset was 40.2 ± 10.8 years, with a mean disease duration of 9.9 ± 6.0 years and an average follow-up of 44.9 months. The mean number of CAG repetitions was 70.9, and the mean SARA score was 11.6. The SCA3 patients were classified into three stages at the first ophthalmological visit: nine patients in stage 1, five in stage 2, and three patients in stage 3.

**Table 1:** Baseline SCA patient characteristics (*n* = 17 cases)

Sex, *n* (%) females	10 (58.8)
Age at onset, mean (SD) [range], years	40.2 (10.8) [25–63]
Disease duration, mean (SD) [range], years	9.9 (6.0) [1.5–22.8]
CAG, mean (SD) [range], number of repetitions	70.9 (2.6) [67–76]
SARA score, mean (SD) [range]	11.6 (5.1) [4-20]
Disease stage	
Stage 1, *n* (%)	9 (52.9)
Stage 2, *n* (%)	5 (29.4)
Stage 3, *n* (%)	3 (17.7)
Follow-up duration, mean (SD) [range], months	44.9 (25.0) [5.3–106.4]
BCVA (logMAR)	0.08 (0.13) [0–0.52]
Structural SD-OCT features	
CMT (µm)	258 (22) [206–295]
MV (mm^3^)	9.99 (0.45) [9.1–10.8]
RNFL (µm), overall	80.0 (6.5) [66–98]
GCC (µm), overall	77.3 (4.9) [70–86]
GCC (µm), minimum	75.6 (5.6) [65–85]

Baseline SCA3 clinical variables—including disease duration, number of CAG repeats, and SARA score—were correlated with structural SD-OCT features (Table 2). The CMT was inversely correlated with disease duration (*r* =  − 0.590; *p* < 0.001) and SARA score (*r* =  − 0.560; *p* = 0.001), and the MV was related to the number of CAG repeats (*r* =  − 0.552; *p* = 0.012). The RNFL thickness was associated with the number of CAG repeats (*r* =  − 0.503; *p* = 0.024) and SARA score (*r* =  − 0.390; *p* = 0.023). In addition, the GCC thickness was related to both the overall and minimum disease duration (*r* =  − 0.585; *p* < 0.001 and *r* =  − 0.612; *p* < 0.001, respectively) and with the number of CAG repeats (*r* =  − 0.493; *p* = 0.027 and *r* =  − 0.513; *p* = 0.021, respectively). The minimum GCC thickness was also related to the SARA score (*r* =  − 0.411; *p* = 0.016). Furthermore, the association of the age at SCA3 diagnosis was analyzed in terms of both clinical (CAG repeats and SARA) and ophthalmological characteristics (CMT, MV, RNFL, GCC). The age of SCA3 onset was indeed correlated with the number of CAG repeats (*r* =  − 0.591; *p* = 0.006), but no other significant associations were found (SARA score *r* = 0.158, *p* = 0.372; CMT *r* =  − 0.032, *p* = 0.857; MV *r* = 0.107, *p* = 0.547; RNFL *r* = 0.194, *p* = 0.271; GCC *r* = 0.001, *p* = 0.997).

**Table 2:** Correlation between baseline clinical characteristics and SD-OCT features^a^

	Disease duration	CAG	SARA
CMT (µm)	− 0.590 (< 0.001)	− 0.287 (0.220)	− 0.560 (0.001)
MV (mm^3^)	− 0.308 (0.077)	− 0.552 (0.012)	− 0.153 (0.387)
RNFL (µm), overall	− 0.304 (0.081)	− 0.503 (0.024)	− 0.390 (0.023)
GCC (µm), overall	− 0.585 (< 0.001)	− 0.493 (0.027)	− 0.301 (0.084)
GCC (µm), minimum	− 0.612 (< 0.001)	− 0.513 (0.021)	− 0.411 (0.016)

^a^Values are the Pearson correlation coefficients (*p* value)

Changes in the clinical variables during follow-up are detailed in Table 3. A significant worsening was found in both the SARA score (+ 34.8%; *p* < 0.001) and BCVA (+ 7.5% logMAR; *p* = 0.047). The disease stage distribution also worsened during follow-up (*p* < 0.001) from mostly stage 1 subjects (52.9%) to mostly stage 3 cases (58.8%). Regarding SD-OCT measurements, the mean CMT decreased (− 2.6%; *p* = 0.026), as did the mean MV (− 1.9%; *p* < 0.001) and the average GCC thickness (− 3.6%; *p* < 0.001). In contrast, no statistically significant change was reported for the overall RNFL thickness (− 1.5%; *p* = 0.113). The rates of change of the clinical variables and SD-OCT features during follow-up (annual rates of change) are noted in Table 3.

**Table 3:** Changes in the clinical characteristics over follow-up

	Baseline	Final follow-up	*p* value	Change (%)
SARA score (points)	11.69 (5.25)	15.75 (5.72)	< 0.001^**a**^	+ 34.8
Disease stage				
Stage 1, *n* (%)	9 (52.9)	5 (29.4)	< 0.001^**b**^	-
Stage 2, *n* (%)	5 (29.4)	2 (11.8)		-
Stage 3, *n* (%)	3 (17.7)	10 (58.8)		-
BCVA (logMAR)	0.08 (0.13)	0.13 (0.17)	0.047^**a**^	+ 7.5
Structural SD-OCT features				
CMT (µm)	258 (22)	252 (27)	0.026^**a**^	− 2.6
MV (mm^3^)	9.99 (0.45)	9.80 (0.52)	< 0.001^**a**^	− 1.9
RNFL (µm), overall	80.0 (6.5)	78.8 (7.2)	0.113^**a**^	− 1.5
GCC (µm), overall	77.3 (4.9)	74.5 (6.2)	< 0.001^**a**^	− 3.6
GCC (µm), minimum	75.6 (5.6)	71.0 (10.5)	0.002^**a**^	− 6.3

Variables shown as the mean (SD)

^a^Paired samples *t* test

^b^Fisher’s exact test

**Fig. 1:**
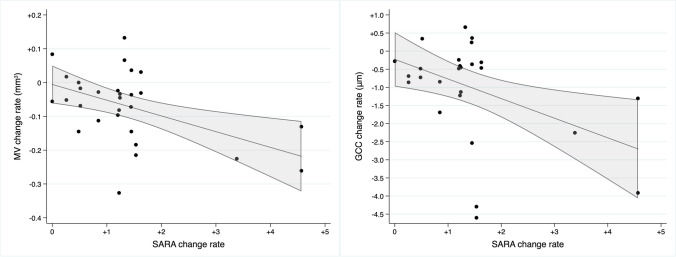
Correlation of annual rates of change of the MV and GCC thickness with the annual rate of change of the SARA score

A correlation study was undertaken between the rates of change of the SCA3 SD-OCT variables and the rate of change of the main clinical variable (SARA score) (Table 4, Fig. 1). The MV annual change rate was inversely associated with the SARA score annual change rate (*r* =  − 0.494; *p* = 0.005). In addition, the annual rate of change of the overall GCC thickness was also related to that of the SARA score (*r* =  − 0.454; *p* = 0.012). On the other hand, regarding the binarized disability stages (low [stages 1 and 2] vs high [stage 3]), logistic regression analysis showed that the annual rate of change of the MV was independently inversely associated with a high disability stage (MV change rate ß coefficient − 19.27; *p* = 0.028).

**Table 4:** Correlation between the rates of change of SD-OCT features and SARA score^a^

	Annual change rate	Correlation between SARA annual rate of change and SD-OCT parameter rates of change	
		Correlation coefficient	*p* value^a^
SARA (points)	+ 1.41 (1.11)	-	-
CMT (µm)	− 1.95 (3.86)	− 0.343	0.058
MV (mm^3^)	− 0.07 (0.10)	− 0.494	0.005
RNFL (µm), overall	− 0.17 (2.63)	− 0.069	0.713
GCC (µm), overall	− 0.94 (1.34)	− 0.454	0.012
GCC (µm), minimum	− 3.16 (12.37)	− 0.298	0.109

Rates of change shown as the mean (SD)

^a^Pearson correlation analysis

## Discussion

This research on longitudinal ophthalmologic assessments in SCA3 patients found that characteristic clinical (SARA score and disease duration) and genetic features (number of CAG repeats) were associated with objective anatomical measurements of the retinal cell layers (CMT, MV, RNFL, GCC, and minimum GCC) from OCT. Furthermore, progression of structural loss, as shown by the rates of decrease of the MV and GCC thickness during follow-up, was associated with worsening of the SARA score. Nevertheless, the annual rate of decrease in the MV seems to be independently associated with a high stage of clinical disability.

Objective retinal and optic nerve fiber involvement in several neurologic disorders has already been described in several reports [3, 10, 16–19]. A decrease in RNFL and GCC thickness was observed in patients with inherited optic nerve disorders, such as dominant optic atrophy [20], and a decrease in the RNFL thickness has been reported in Leber’s hereditary optic neuropathy [21]. In addition, regarding neurodegenerative diseases, RNFL and GCC thinning have both been found in patients with Alzheimer’s and Parkinson’s disease, even in early stages of the disorder without visual acuity loss [10, 16]. Regarding neurodegenerative ataxia, a decrease in the RNFL and GCC layer and overall macular thickness has been reported in Friedreich ataxia patients [22]. In terms of SCA, previous reports [23] have used OCT to investigate SCA1, SCA2, SCA3, and cerebellar multisystem atrophy. They found RNFL thinning in SCA2 and SCA3 but not in SCA1. In contrast, a different study found a decreased average RNFL thickness in a group of SCA1 patients compared with a healthy control group [24]. Recently, the GCC layer was found to be affected in patients with SCA3 relative to healthy controls [5]. They also correlated SD-OCT parameters with the clinical phenotype and found a negative correlation between the SARA and International Cooperative Ataxia Rating Scale (ICARS) scores and the thicknesses of the ganglion cell layer and inner plexiform layer. Finally, in a comparative OCT study [25] evaluating patients with SCA10 and SCA3, a negative correlation was found between SARA and RNFL thickness, but only in the nasal area of the peripapillary disc.

In the present study, we assessed the associations of characteristic clinical variables of SCA3, such as disease duration, the number of CAG repeats, and the SARA score, with objective anatomical changes using SD-OCT. Regarding specific SD-OCT parameters, RNFL thickness was found to be significantly correlated with the number of CAG repeats and the SARA score. The analysis of GCC thickness revealed a significant correlation with disease duration and the number of CAG repeats. These associations can help physicians assess the severity of the disease using a noninvasive method. As SCA3 is a progressive neurodegenerative disease, changes in both clinical variables and OCT features will emerge over its course. Therefore, the analysis of the relationship between the annual rate of retinal cell layer loss and the progression of the clinical status of the disease produced interesting results. Thus, a progressive decrease in the MV and GCC thickness in this SD-OCT study was directly associated with an annual worsening of SCA3 symptoms (increase in the SARA score). In addition to the SARA score, the binarized clinical disability stage was also analyzed, revealing that the MV was independently associated with the high disability stage of the disease.

The reported relations imply that more severe clinical disease features—greater SARA score and disability stage—could indeed be associated with objective and progressive loss of retinal cell layers as determined by SD-OCT. Therefore, objective outcomes such as MV and GCC thickness measurements could be considered biomarkers of SCA3 disease to some extent. Importantly, the associations of MV should be carefully interpreted, as it encompasses the GCC layer itself, meaning that MV changes could be intrinsically associated with GCC loss. A previous study described OCT and electroretinography (ERG) findings as potential biological markers of disease progression and severity in patients with SCA3, suggesting the possibility of photoreceptor involvement [26]. Taken together, these findings are considered to have strong diagnostic value and could have extensive use in clinical practice. Furthermore, given the difficulties in exploring the advanced stages of SCA3, rapid and objective noninvasive imaging with clinical correlation could be of great importance.

Interestingly, the number of CAG repeats is not always reported in the literature. Since the phenotype in SCA3 patients varies widely depending on the number of CAG repeats, such information should be considered [27]. In the present study, we found a significant inverse correlation between the number of CAG repeats and SD-OCT parameters such as the MV, RNFL, and GCC. This leads us to consider that worse OCT parameters are likely to be detected in the most severe forms of the disease, that is, those harboring a higher number of CAG repeats. This is consistent with the current clinical understanding of SCA3 and therefore strengthens the external validity of the presented results.

The pathophysiology of visual pathway cell degeneration in SCA3 seems to be similar to that observed in Friedreich ataxia, with a decrease/impairment in the thickness of RNFL and GCC and a reduction in the MV [22]. A decrease in macular thickness measured on OCT has also been observed in dominant optic atrophy due to the degeneration of retinal ganglion cells of the papillomacular bundle, which is thought to subsequently drive RNFL loss [20]. On the other hand, the cause of RNFL loss in Alzheimer’s and Parkinson’s disease is thought to have a multifactorial ethology, including degeneration of the GCC and retrograde degeneration caused by the loss of cortical neurons [8, 28]. Therefore, based on our results in SCA3 patients, RNFL and GCC loss could also be considered to have a multifactorial ethology, partially explained by retrograde degeneration, in a similar way to Alzheimer’s disease [8].

This study has some limitations that should be disclosed. Anatomical parameters measured by OCT are an excellent tool for obtaining objective measurements. However, due to the small number of cases and the absence of a control group, the reported changes and associations in SD-OCT measurements may not be entirely free of biases. For instance, regarding age at SCA3 onset, younger patients are expected to present with more severe forms of the disease and ocular involvement. Such a correlation was indeed reported in our study, in which age at onset was inversely associated with the number of CAG repeats but not with other clinical and ophthalmological features. However, this must be carefully interpreted considering our small cohort with great dispersion of age at diagnosis and kept within a pilot study nature. Due to the observational nature of the present study, a strategy for multiplicity adjustment was not fully planned; thus, the results should be validated in future independent studies. Finally, additional explorations such as electrophysiological testing and low contrast visual acuity could have been useful for acquiring more sensitive information on visual dysfunction in these patients. In summary, and within the limitations of a proof-of-concept approximation of a very rare disease, we consider the results and conclusions of this study to be of notable importance.

## Conclusion

Advanced analysis of SD-OCT images of the eye has recently emerged as a new tool for correlating the clinical features of SCA3 patients with objective anatomical measurements. Therefore, progressive and repetitive OCT-based measurements of the retinal layers could be considered disease biomarkers of great importance given the reported relationships with the clinical features of SCA3. Accordingly, MV and GCC thickness loss rates seem to be the most reliable biomarkers in this regard. Physicians could enhance the everyday follow-up capabilities for these difficult and rare Machado-Joseph disease patients with an objective approach that emphasizes the relationship between retinal structure and cerebellar signs.

## Data Availability

The data used in this study are available upon request from the authors due to privacy and confidentiality considerations.
